# Adjuvant radiotherapy after curative surgery for oral cavity squamous cell carcinoma and treatment effect of timing and duration on outcome—A Taiwan Cancer Registry national database analysis

**DOI:** 10.1002/cam4.1611

**Published:** 2018-06-14

**Authors:** Yung‐Jen Cheng, Mu‐Hung Tsai, Chun‐Ju Chiang, Sen‐Tien Tsai, Tsang‐Wu Liu, Pei‐Jen Lou, Chun‐Ta Liao, Jin‐Ching Lin, Joseph Tung‐Chieh Chang, Ming‐Hsui Tsai, Pen‐Yuan Chu, Yi‐Shing Leu, Kuo‐Yang Tsai, Shyuang‐Der Terng, Chih‐Yen Chien, Muh‐Hwa Yang, Sheng‐Po Hao, Chuan‐Cheng Wang, Ming‐Hsun Tsai, Helen H. W. Chen, Chin Kuo, Yuan‐Hua Wu

**Affiliations:** ^1^ Department of Radiation Oncology National Cheng Kung University Hospital College of Medicine National Cheng Kung University Tainan Taiwan; ^2^ Institute of Epidemiology and Preventive Medicine College of Public Health National Taiwan University Taipei Taiwan; ^3^ Taiwan Cancer Registry Taipei Taiwan; ^4^ Department of Otolaryngology National Cheng Kung University Hospital College of Medicine National Cheng Kung University Tainan Taiwan; ^5^ National Institute of Cancer Research National Health Research Institutes Taipei Taiwan; ^6^ Department of Otolaryngology National Taiwan University Hospital and College of Medicine Taipei Taiwan; ^7^ Department of Otorhinolaryngology, Head and Neck Surgery Chang Gung Memorial Hospital Chang Gung University Taoyuan Taiwan; ^8^ Department of Radiation Oncology Taichung Veterans General Hospital Taichung Taiwan; ^9^ Department of Radiation Oncology, Head and Neck Oncology Group Chang Gung Memorial Hospital Chang Gung University Taoyuan Taiwan; ^10^ Department of Otorhinolaryngology, Head and Neck Surgery China Medical University Hospital Taichung Taiwan; ^11^ Department of Otolaryngology Taipei Veterans General Hospital Taipei Taiwan; ^12^ Department of Otolaryngology Mackay Memorial Hospital Taipei Taiwan; ^13^ Department of Oral and Maxillofacial Surgery, Head and Neck Surgery Changhua Christian Hospital Changhua Taiwan; ^14^ Department of Head and Neck Surgery Koo Foundation Sun Yat‐Sen Cancer Center Taipei Taiwan; ^15^ Department of Otolaryngology Chang Gung Memorial Hospital – Kaohsiung Medical Center Chang Gung University College of Medicine Kaohsiung Taiwan; ^16^ Division of Medical Oncology Department of Oncology Taipei Veterans General Hospital Taipei Taiwan; ^17^ Department of Otolaryngology Head and Neck Surgery Shin Kong Wu Ho‐Su Memorial Hospital Taipei Taiwan; ^18^ Division of Medical Oncology in the Hematology‐Oncology Department of Internal Medicine Changhua Christian Hospital Changhua Taiwan

**Keywords:** adjuvant radiotherapy, cancer database, oral cancer, prognostic factor, treatment time

## Abstract

Conduct an accurate risk assessment of resected oral cavity squamous cell carcinoma (OSCC) patients by accessing a nationwide systemic investigation is pivotal to improve treatment outcomes. In this article, we tried to determine the impact of different prognostic factors for OSCC patients who received adjuvant radiotherapy (RT) after curative surgery, using Taiwan's national cancer registry database (TCR). A nationwide, large population‐based study was conducted using TCR with patients identified from 2007 to 2015. The study variables included age, gender, cancer subsites, stage, histology grade, margin and extra‐nodal extension (ENE) status, treatment type, surgery to RT interval (ORI), total RT treatment time (RTT), and RT dose. Univariate and multivariate analysis were performed to identify predictors of the variables associated with overall survival (OS), cause‐specific survival (CSS), local‐regional relapse‐free survival (LRFS), and distant metastasis‐free survival (DMFS). 8986 OSCC patients treated with surgery and adjuvant RT were analyzed. In multivariate analysis, worse outcomes were associated with males, older age, subsite in the oral tongue, advanced stage, higher histologic grade, involved margin, and positive ENE. ORI only showed an adverse trend in LRFS, when exceeding 7 weeks (*P* = .06). RTT >8 weeks was a significant poor predictor in OS, CSS and LRFS (*P* < .001). Extreme RT dose (>70 Gy or ≤50 Gy) also demonstrated an adverse impact on the outcomes. Prolonged RT treatment time and extreme RT doses were identified as significantly poor prognostic predictors in OSCC patients who received adjuvant RT after curative surgery.

## 1 INTRODUCTION

In Taiwan, the incidence of oral cancer is high (21.54/100 000 in 2014) and it accounts for nearly 70% of newly diagnosed head and neck cancers. In 2014, it was the fifth leading cause of cancer death noted in the Taiwan Cancer Registry Annual Report.^1^ Generally, radical surgery remains the main treatment for patients with resectable oral cavity squamous cell carcinoma (OSCC). In several clinical trials, adjuvant radiotherapy with or without chemotherapy in patients with unfavorable pathological findings has been shown to improve disease control and survival.^2^, ^3^ Although combinations of therapeutic protocols have improved the outcomes and quality of life, the mortality and morbidity rates of resected OSCC are still high.

OSCC is a multifactorial disease. The associated research literature has focused on searching for independent factors or groups of factors which impact on prognosis for OSCC patients.^4^, ^5^ However, most studies were of small sample sizes or mixed head and neck cancer entities and not exclusively limited to OSCC, which may have contributed to difficulties in interpretation of data. Additionally, some factors remain controversial and there are ambiguous results for outcomes in historical series. It is therefore pivotal to conduct an accurate risk assessment of site specific OSCC patients by accessing a systemic investigation to improve treatment outcomes.

The nationwide Taiwan Cancer Registry database (TCR), which has been run for over 30 years by the Health Promotion Administration, Ministry of Health and Welfare, Taiwan, has provided much of the essential foundation for academic research and cancer control policy in Taiwan. Using the database, we sought to create more predictive, preventive, personalized, and participatory healthcare.^6^ Since 2002, the TCR database has included a newer dataset of detailed information on cancer stages, treatment approaches, and tumor relapses, achieving more refined record of patient status. Until now, this database collects data from all the major hospitals in Taiwan and captures an estimated over 98% of newly diagnosed OSCC cases.

In this article, we tried to raise a large population‐based study by examining the impact of different factors in prognosis for OSCC treated with radical surgery and adjuvant RT using the national Taiwan Cancer Registry database.

## 2 PATIENTS AND METHODS

### 2.1 Selection of patients

**Figure 1:**
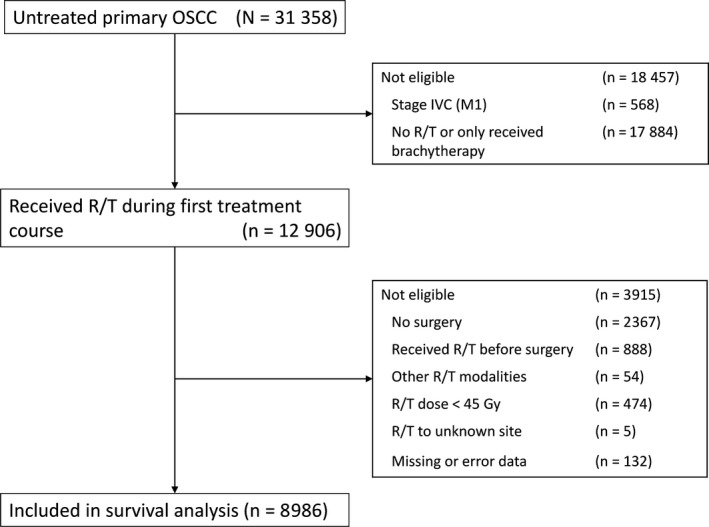
CONSORT diagram of the patients selection through the study

Using the TCR database, 31 358 patients diagnosed with OSCC between 2007 and 2015 were identified. The enrolled oral cavity cancer subsites included lip cancer (C00.0; C00.1; C00.2; C00.3; C00.4; C00.5; C00.6; C00.8; C00.9), tongue cancer (C02.0; C02.1; C02.2; C02.3; C02.8; C02.9), gingival cancer (C03.0; C03.1; C03.9), floor of mouth cancer (C04.0; C04.1; C04.8; C04.9), hard palate cancer (C05.0; C05.8; C05.9), buccal cancer (C06.0; C06.1; C06.2), and other forms of oral cancer (C06.8; C06.9) [ICD‐O‐3 codes (International Classification of Diseases for Oncology, third Edition)]. Patients receiving adjuvant RT with/without chemotherapy after curative surgery were included. Exclusion criteria included previous cancer and RT history before OSCC diagnosed, nonsquamous histology, initial metastatic disease (stage IVC) and patients who receiving brachytherapy only. Those missing data on any of the variables and with unknown recurrent site were also excluded. To minimize the effect of the RT dose, we only included patients receiving an RT dose of 45 Gy or more. All data was validated till the end of 2016. A CONSORT diagram illustrating the cohort selection is provided in Figure 1. The study was reviewed and determined to have exempt status by the Research Ethics Committee of the National Health Research Institutes (EC1050804‐E).

### 2.2 Study variables

The study variables, including age at diagnosis, gender, oral cavity subsites, histology grade, AJCC stage, treatment type, margin status, ORI, RTT, and ENE status were all collected from the TCR database. Tumor staging was according to the American Joint Committee on Cancer (AJCC), sixth and seventh Edition staging guidelines.^7^, ^8^ Data on the ENE status was only available from 2011 onwards, when this data was first included in collection for the national dataset. Consequently, ENE variables are made in another analysis. ORI was defined as the time interval between the day of surgery and initiation of adjuvant radiotherapy, and RTT was defined as the overall RT treatment time period. The primary outcomes were overall survival (OS) and local‐regional free survival (LRFS). By virtue of access to existing data, cancer‐specific survival (CSS) and distant‐metastasis free survival (DMFS) were also analyzed, as recurrence and cause of death are also recorded in the TCR database.^6^

### 2.3 Statistical analysis

All survival times were calculated from the date of surgery. Cumulative survival plots for the study endpoints with different variables were evaluated with univariate analysis using the Kaplan‐Meier method. To test statistically significant differences between curves, the log‐rank test was used. All the variables included in the univariate analysis are also included in the final multivariate model as the statistical specialist suggested. The multivariate analysis using the Cox proportional hazards regression model was performed to identify covariates that were significantly associated with the aforementioned endpoints (OS, CSS, LRFS, and DMFS). All results were expressed as hazard ratios (HRs) with 95% confidence intervals (CIs). All statistical analysis was performed using SAS software, version 9.4 (SAS Institute Inc., Cary, NC). Two‐tailed *P* values <.05 were considered statistically significant. The first failure pattern results of all patients in the study were also roughly calculated.

## 3 RESULTS

### 3.1 Patient characteristics and initial treatment approach

Patient characteristics were summarized in Table 1. From 2007 to 2015, 8986 patients with primary OSCC receiving adjuvant radiotherapy after curative surgery were analyzed. At the end of 2016, there were 5508 (61.3%) surviving patients and the median follow‐up time among them was 53.4 months (range 12.0‐119.7 months). Patient age ranged from 19 to 91 years (median: 52). The population was predominantly male with 8310 (92.5%) males, and 676 (7.5%) females (male/female ratio 12.3:1). The most common subsite of the population was oral tongue (n = 3213, 35.8%), followed by buccal mucosa (n = 2767, 30.8%), gum (n = 1414, 15.7%), floor of mouth (n = 389, 4.3%), lip (n = 221, 2.5%) and hard palate (n = 206, 2.3%). The proportions of pathological stages for stages I, II, III, and IV were 5.6%, 11.8%, 19.0%, and 63.7%, respectively. Pathological positive margin after surgery was documented in 831 (9.2%) patients. ENE data was retrieved only from 2011 to 2015 with a total number of 5475 (negative n = 4048, 45.0% and positive n = 1427, 15.9%) (Table 1). Treatment parameters including treatment type, ORI, RTT, and RT dose were also summarized in Table 1.

**Table 1:** Characteristics of patients (n = 8,986; 2007‐2015)

Covariate		No. of cases	Percent
Gender	Male	8310	92.5
	Female	676	7.5
Age	<40	870	9.7
	40‐49	2643	29.4
	50‐59	3219	35.8
	60‐69	1627	18.1
	≧70	627	7.0
Subsite	Buccal mucosa	2767	30.8
	Lip	221	2.5
	Oral tongue	3213	35.8
	Gum	1414	15.7
	Floor of mouth	389	4.3
	Hard palate	206	2.3
	Other parts of mouth	776	8.6
AJCC Stage	Stage I	503	5.6
	Stage II	1056	11.8
	Stage III	1703	19.0
	Stage IV (M0)	5724	63.7
Differentiation	Well	2145	23.9
	Moderate	5653	62.9
	Poorly or undifferentiated	1069	11.9
	Unknown	119	1.3
Margin status	Negative	8155	90.8
	Positive	831	9.2
Extranodal extension	Negative	4048	45.0
	Positive	1427	15.9
	Unknown (before 2011)	3511	39.1
Treatment type	Surgery ‐> RT	2865	31.9
	Surgery ‐> CCRT	5546	61.7
	Surgery ‐> RT + CT	418	4.7
	CT ‐> Surgery ‐> RT	157	1.7
OP‐RT interval	ORI ≤ 4 wk	1704	19.0
	4 < ORI ≤5 wk	2243	25.0
	5 < ORI ≤6 wk	2769	30.8
	6 < ORI ≤7 wk	924	10.3
	ORI > 7 wk	1346	15.0
RT time	RTT ≤6 wk	1621	18.0
	6 < RTT ≤7 wk	4574	50.9
	7 < RTT ≤8 wk	1790	19.9
	RTT > 8 wk	1001	11.1
RT dose	45 <= Dose <= 50 Gy	130	1.4
	50 < Dose <= 60 Gy	2308	25.7
	60 < Dose <= 70 Gy	6115	68.1
	Dose > 70 Gy	433	4.8

AJCC, American joint committee on Cancer; CCRT, concurrent chemoradiation therapy; CT, chemotherapy; OP, operation; ORI, OP‐RT interval; RT, radiotherapy; RTT, RT treatment time.

### 3.2 Patient and disease characteristics related to survival analysis

**Figure 2:**
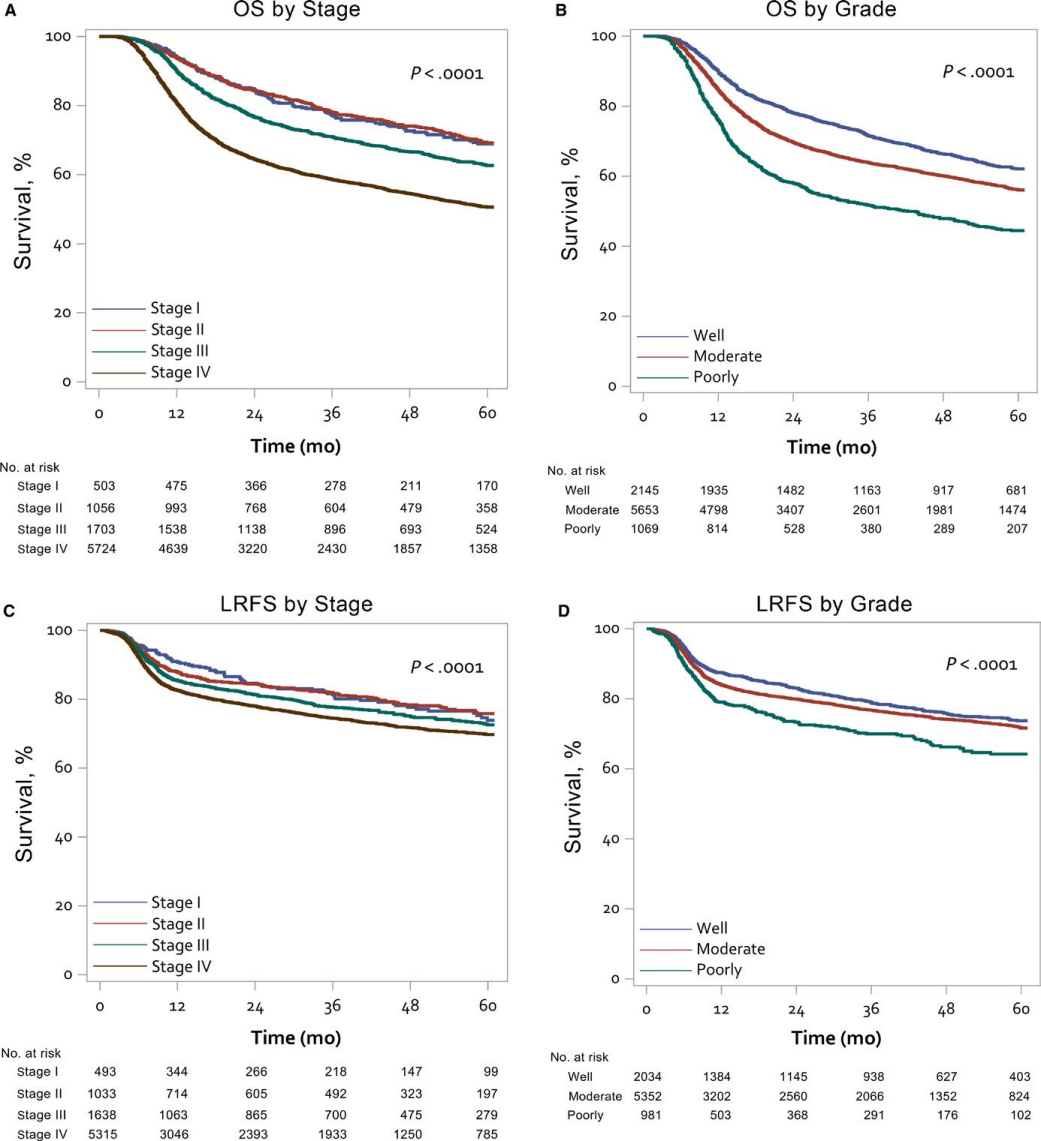
Kaplan‐Meier plots of the 5‐y overall survival (OS) and local‐regional free survival (LRFS) stratified by disease characteristics. OS according to cancer stage (panel A) and according to tumor grade (panel B); LRFS according to cancer stage (panel C) and according to tumor grade (panel D)

The Kaplan‐Meier survival curves of 5‐year OS and LRFS according to tumor stage and grade were shown in Figure 2. Univariate analysis of the patient and disease characteristics for the OS, CSS, LRFS, and DMFS rate are shown in Table S1. In the multivariate analysis, age >70, subsite of oral tongue, stage III or IV, higher histological grade and surgical margin were all identified as poor prognostic factors for both the OS and CSS. Male gender showed a statistically significant adverse impact not only on the OS but on CSS. In multivariate analysis of LRFS, male, stage III or IV, higher grade and surgical margin all significantly impacted the outcome, while stage III or IV and higher grade were significant in DMFS (Table 2). In analysis of patients from 2011, ENE positive also showed significant impacts on all the survival outcomes (*P* < .001) in multivariate analysis. An additional supplement, which outlines not much decisive change after incorporating the ENE into multivariate analysis with the data all retrieving after the year of 2011, is demonstrated in Table S3.

**Table 2:** Multivariate analysis of factors associated with each endpoints

Patient characteristics	OS		CSS		LRFS		DMFS	
	HR (95% CI)	*P*‐value	HR (95% CI)	*P*‐value	HR (95% CI)	*P*‐value	HR (95% CI)	*P*‐value
Sex								
Male	Ref		Ref		Ref		Ref	
Female	**0.85 (0.74‐0.97)**	**.020**	0.93 (0.81‐1.08)	.359	**0.82 (0.67‐0.99)**	**.045**	0.89 (0.67‐1.18)	.443
Age								
<40	Ref		Ref		Ref		Ref	
40‐49	0.97 (0.86‐1.10)	.621	0.95 (0.83‐1.08)	.416	1.01 (0.86‐1.19)	.938	0.92 (0.73‐1.17)	.507
50‐59	0.95 (0.84‐1.07)	.411	0.89 (0.78‐1.01)	.077	0.92 (0.78‐1.08)	.316	0.84 (0.67‐1.07)	.159
60‐69	1.09 (0.96‐1.25)	.198	0.96 (0.83‐1.11)	.588	0.83 (0.69‐1.00)	.050	**0.70 (0.53‐0.92)**	**.011**
>70	**1.48 (1.26‐1.73)**	**<.001**	**1.22 (1.02‐1.46)**	**.026**	0.84 (0.66‐1.06)	.142	0.94 (0.66‐1.34)	.746
Disease characteristics								
Site of disease								
Buccal mucosa	Ref		Ref		Ref		Ref	
Lip	0.92 (0.72‐1.15)	.469	0.94 (0.72‐1.20)	.639	0.94 (0.68‐1.26)	.679	0.77 (0.44‐1.25)	.321
Oral tongue	**1.10 (1.01‐1.19)**	**.027**	1.06 (0.97‐1.16)	.202	0.98 (0.88‐1.10)	.794	0.99 (0.83‐1.17)	.899
Gum	**0.86 (0.77‐0.96)**	**.005**	**0.85 (0.75‐0.95)**	**.006**	0.88 (0.75‐1.02)	.08	0.85 (0.68‐1.06)	.151
Floor of Mouth	0.95 (0.79‐1.12)	.541	0.82 (0.66‐1.00)	.052	**0.45 (0.32‐0.62)**	**<.001**	1.00 (0.70‐1.39)	.993
Hard palate	1.16 (0.94‐1.42)	.160	1.15 (0.91‐1.44)	.232	1.29 (0.96‐1.69)	.081	0.93 (0.54‐1.48)	.766
Other parts of Mouth	1.07 (0.94‐1.21)	.305	1.03 (0.90‐1.18)	.655	1.10 (0.93‐1.30)	.271	0.92 (0.70‐1.19)	.531
Pathologic AJCC stage								
Stage1	Ref		Ref		Ref		Ref	
Stage2	1.03 (0.84‐1.28)	.778	1.08 (0.84‐1.39)	.557	1.09 (0.85‐1.41)	.501	1.80 (0.79‐4.87)	.199
Stage3	**1.41 (1.16‐1.72)**	**<.001**	**1.51 (1.21‐1.91)**	**<.001**	**1.32 (1.04‐1.68)**	**.023**	**4.17 (1.99‐10.75)**	**<.001**
Stage4	**2.10 (1.76‐2.54)**	**<.001**	**2.44 (1.98‐3.05)**	**<.001**	**1.55 (1.24‐1.95)**	**<.001**	**9.19 (4.49‐23.37)**	**<.001**
Grade								
Well differentiated	Ref		Ref		Ref		Ref	
Moderately differentiated	**1.26 (1.16‐1.38)**	**<.001**	**1.26 (1.15‐1.39)**	**<.001**	**1.16 (1.04‐1.31)**	**.010**	**1.75 (1.43‐2.15)**	**<.001**
Poorly or Undifferentiated	**1.81 (1.61‐2.03)**	**<.001**	**1.84 (1.62‐2.08)**	**<.001**	**1.59 (1.35‐1.87)**	**<.001**	**2.55 (1.99‐3.28)**	**<.001**
Unknown	**1.33 (0.99‐1.75)**	.051	**1.43 (1.04‐1.92)**	**.021**	**1.57 (1.07‐2.23)**	**.015**	0.95 (0.40‐1.90)	.897
Margin								
Negative	Ref		Ref		Ref		Ref	
Positive	**1.60 (1.44‐1.77)**	**<.001**	**1.61 (1.44‐1.80)**	**<.001**	**1.47 (1.25‐1.71)**	**<.001**	1.13 (0.88‐1.44)	.318
Treatment parameters								
Treatment type								
OP‐>RT	Ref		Ref		Ref		Ref	
OP‐>CCRT	1.07 (0.98‐1.16)	.146	1.10 (1.00‐1.21)	.052	**0.83 (0.74‐0.93)**	**.001**	**1.55 (1.27‐1.91)**	**<.001**
OP‐>RT + CT	1.03 (0.87‐1.21)	.743	1.04 (0.86‐1.25)	.655	0.81 (0.63‐1.01)	.072	1.13 (0.74‐1.68)	.550
CT‐>OP‐>RT	1.15 (0.89‐1.46)	.265	**1.30 (0.99‐1.68)**	**.049**	0.95 (0.67‐1.32)	.775	1.52 (0.85‐2.53)	.132
OP to RT interval (ORI)								
0‐4 wk	Ref		Ref		Ref		Ref	
4‐5 wk	0.92 (0.83‐1.01)	.088	0.92 (0.82‐1.03)	.155	0.97 (0.84‐1.12)	.672	1.13 (0.92‐1.40)	.244
5‐6 wk	0.92 (0.83‐1.01)	.084	0.92 (0.83‐1.03)	.147	0.92 (0.81‐1.06)	.256	1.09 (0.89‐1.33)	.429
6‐7 wk	0.98 (0.86‐1.11)	.737	0.95 (0.83‐1.09)	.487	1.05 (0.88‐1.25)	.552	1.03 (0.78‐1.35)	.823
>7 wk	1.07 (0.96‐1.20)	.209	1.09 (0.97‐1.24)	.148	1.16 (0.99‐1.35)	.061	0.98 (0.76‐1.26)	.853
RT treatment time (RTT)								
0‐6 wk	Ref		Ref		Ref		Ref	
6‐7 wk	0.95 (0.85‐1.07)	.436	0.97 (0.86‐1.11)	.685	1.02 (0.87‐1.20)	.806	0.94 (0.73‐1.22)	.642
7‐8 wk	1.12 (0.98‐1.27)	.102	1.14 (0.98‐1.32)	.086	**1.28 (1.07‐1.53)**	**.008**	1.11 (0.84‐1.48)	.479
>8 wk	**1.40 (1.21‐1.61)**	**<.001**	**1.46 (1.25‐1.70)**	**<.001**	**1.57 (1.28‐1.91)**	**<.001**	0.93 (0.67‐1.29)	.660
RT dose								
45‐50 Gy	**2.02 (1.57‐2.56)**	**<.001**	**2.00 (1.50‐2.62)**	**<.001**	1.44 (0.93‐2.13)	.085	1.46 (0.66‐2.82)	.299
50‐60 Gy	Ref		Ref		Ref		Ref	
60‐70 Gy	**1.11 (1.00‐1.23)**	**.042**	**1.13 (1.01‐1.27)**	**.039**	1.08 (0.95‐1.25)	.250	**1.28 (1.02‐1.62)**	**.039**
>70 Gy	**1.28 (1.08‐1.52)**	**.004**	**1.34 (1.11‐1.61)**	**.002**	**1.33 (1.05‐1.68)**	**.015**	**1.72 (1.19‐2.47)**	**.004**

AJCC, American joint committee on Cancer; CCRT, concurrent chemoradiation therapy; CI, confidence interval; CT, chemotherapy; CSS, cancer‐specific survival; DMFS, distant‐metastasis free survival; HR, hazard ratio; LRFS, local‐regional free survival; OP, operation; ORI, OP‐RT interval; OS, overall survival; RT, radiotherapy; RTT, RT treatment time.

Boldface indicates *P* < .05

### 3.3 Treatment parameters related to survival analysis

**Figure 3:**
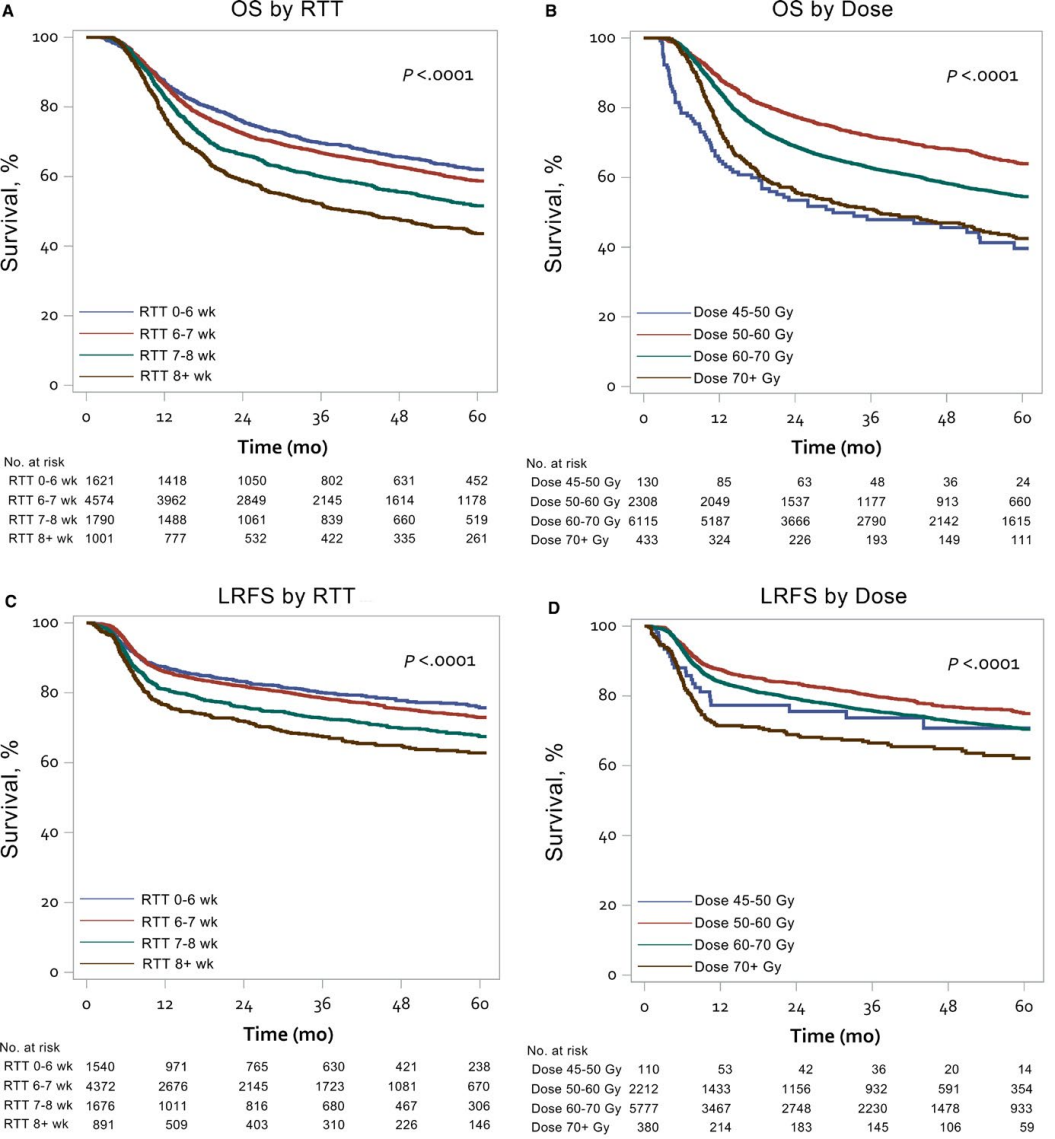
Kaplan‐Meier plots of the 5‐y overall survival (OS) and local‐regional free survival (LRFS) stratified by treatment parameters. OS according to radiotherapy treatment time (panel A) and according to radiotherapy dose (panel B); LRFS according to radiotherapy treatment time (panel C) and according to radiotherapy dose (panel D)

The Kaplan‐Meier survival curves of 5‐year OS and LRFS according to RTT and RT dose were shown in Figure 3. In the multivariate analysis of treatment parameters and survival, treatment by adjuvant CCRT after surgery was identified as a good predictor in LRFS (HR: 0.83; 95% CI: 0.74‐0.93; *P* < .01). For ORI, only a waiting time over 7 weeks showed a tendency toward having an adverse effect on LRFS (HR: 1.16; 95% CI: 0.99‐1.35; *P* = .06). The adverse effect of RTT gradually increased where treatment time exceeded 7 weeks. RTT between 7 and 8 weeks had significantly detrimental effects on LRFS (HR: 1.28; 95% CI: 1.07‐1.53; *P* < .01) while RTT >8 weeks compromised not only the LRFS (HR: 1.57; 95% CI: 1.28‐1.91; *P* < .001) but also the OS (HR: 1.40; 95% CI: 1.21‐1.61; *P* < .001) and CSS (HR: 1.46; 95% CI: 1.25‐1.70; *P* < .001). Neither ORI nor RTT were significantly related to DMFS (Table 2).

An RT dose ranging from 50 to 60 Gy had the best OS, followed by a range between 60 and 70 Gy. A dose less than 50 Gy or higher than 70 Gy, had the largest negative impact on both OS and CSS. Furthermore, a dose higher than 70 Gy also showed a significant negative impact on LRFS and DMFS (Table 2).

### 3.4 Disease failure pattern

Two thousand four hundred and ten patients (26.8%) had a documented relapse of disease. In these patients, local and regional relapse without metastasis was coded as the first failure site in 1602 cases (17.8%) while distant metastasis only was coded in 593 cases (6.6%). Simultaneous local/regional and metastasis was coded in 215 cases (2.4%). Detailed results of first failure pattern are listed in Table S2.

## 4 DISCUSSION

This study, to the best of our knowledge, analyzed the largest patient cohort yet, as it included 8986 patients with primary resected OSCC receiving adjuvant RT to find the impact of different factors in prognosis. Unlike most of the historical analysis of mixed head and neck cancer, we focused on a homogeneous patient series with primary resected OSCC, followed by adjuvant RT, to avoid confounding interaction between different origins of head and neck cancer.

ENE and positive surgical margins were both identified as major risks to survival in our results. These findings were compatible with most of the results in previous head and neck studies, and the conclusions drawn by the vast majority of authors.^9^, ^10^ Also, greater age was thought to be associated with poorer survival outcomes in many studies.^11^, ^12^ Our study demonstrated a poorer outcome in age >70 groups, and this was generally in line with the poor health condition, complex comorbidities, more likelihood of being in an advanced stage and poor tolerance of treatment in these patients.

In our study, males showed worse survival rates on OS and LRFS than female patients. Honorato et al^13^ demonstrated a lower cumulative 5‐year OS in males by examining 477 resected OSCC patients, although the difference didn't achieve statistical significance (*P* = .25). Similarly, Garavello et al reviewed 142 male and 71 female patients with OSCC. More cancer‐related deaths were observed in men (39%) than in women (32%) (OR: 0.76; 95% CI: 0.42‐1.38), however, the 5‐year PFS and OS were similar (*P* = .31 and *P* = .34).^14^ Although gender did not seem to be a significant determinant of survival in these studies, which could be accounted for by the relatively small study population, our large group analysis reinforced that males have a poorer prognosis for survival.

Subsites for oral cancer have been recognized as a prognostic factor in some studies. Leite et al^12^ demonstrated a higher mortality rate in tongue cancer than in lip cancer (mean survival time 25.4 vs 44.6 months; *P* < .01). Data from 11 cancer registries in Germany also showed a better survival for lip cancer, than for patients with tongue cancer (86.5% vs 48.1%).^15^ We also found that worse outcomes were observed in oral tongue subsites, and a better prognosis in gums, compared to other subsites. This could be explained by rich lymphatic drainage and relatively difficult access in evaluation of the oral tongue over other OSCC subsites.

The histologic grading system was not incorporated into the current staging criteria because of its failure to predict prognosis in some historical data.^16^ In contrast, several studies demonstrated that histologic grade was a significant prognostic predictor which was better than tumor size, nodal status, or margin status.^17^, ^18^ In this study, the histologic grade revealed statistically significant impact in all of the outcomes, much as the AJCC staging system did. Thus, histologic grade should be considered essential additionally to the staging system, when making clinical decisions.

The overall treatment time, which included the interval between surgery and postoperative RT (ORI) and the duration of whole RT course (RTT), showed significant impact on both local control and survival in advanced head and neck cancer patients treated with surgery and RT in a randomized trial.^19^ In that trial, the 5‐year actuarial LRC rates for <11 weeks vs 11‐13 weeks vs >13 weeks were 76%, 62% and 38% (*P* = .002) and the survival rates were 48%, 27%, and 25%, respectively (*P* = .03). The author suggested the entire treatment duration should be controlled to take less than 13 weeks. Using this, our study further illuminated the impact on the outcomes by investigating ORI and RTT separately.

Systematic review of ORI showed a significantly higher local recurrence rate in postoperative head and neck cancer patients whose ORI was over 6 weeks vs those less than 6 weeks (odds ratio = 2.89; 95% CI, 1.60‐5.21).^20^ The authors then concluded that the interval between surgery and start of RT should be as short as possible. The National Comprehensive Cancer Network (NCCN) then took the recommendation that the interval between resection and postoperative RT should be ≤6 weeks. Conversely, in a case series conducted by the Memorial Sloan Kettering Cancer Center, accessing 111 advanced head and neck cancer patients treated with surgery followed by adjuvant RT, there was no significant difference in loco‐regional control between patients ORI ≤6 weeks and ORI > 6 weeks (*P* = .11). Even with analysis which divided the ORI cutoff points at 4, 5, 7 to 11 weeks, no significant results were found.^21^ A recent National Cancer Database (NCDB) analysis also studied the impact of different timeframe of ORI on overall survival by accessing 41 291 head and neck SCC patients who undergoing surgery and adjuvant RT. They concluded that there was no survival benefit to start RT earlier within 6 weeks, but only small progressive survival decrements were noted once ORI beyond 7 weeks.^22^ Our result of ORI was compatible with the NCDB analysis, which showed no significant effects within the recommended 6‐week timeframe.

As for RTT, several studies demonstrated that protracted radiation treatment time would compromise outcomes due to concern about cancer cell repopulation. Withers et al^23^ provided the first clinical evidence that if duration of RTT was prolonged in head and neck cancer, an average increase dose of 0.6 Gy/day was required to compensate for the observed local control loss. Fowler et al^24^ conducted a systemic review for head and neck cancer patients treated with definitive RT and demonstrated a median value of 14% (range 3%‐25%) of local control loss per week of RTT. In the postoperative setting, a retrospective series also demonstrated that a shorter RTT (<6 weeks) was found to be associated with higher rates for LRC (*P* = .0004), DFS (*P* = .0029), and OS (*P* = .006) in resected OSCC patients.^25^

Intriguingly, in our multivariate analysis, ORI did not seem to be an obviously significant prognostic factor, while RTT did show a significant impact on the endpoints when RTT >7 weeks. It could be explained by the finding that tumor clonogens specifically accelerated their proliferation once RT was implemented, which affected the outcomes in squamous cell carcinoma of the head and neck. This phenomenon has been shown in both literature overviews^23^ and in randomized controlled trials.^26^ Furthermore, by means of the improvements in radiation technique, risks associated with delays in initiating RT might be mitigated. Taken together, there would be more flexibility to get better control of the postoperative issues (eg poor wound healing, postoperative complication, or infection) before starting adjuvant RT in recent era. However, unreasonable delay should still be avoided as there was a trend of adverse impact on LRFS when ORI was delayed for more than 7 weeks. Once the RT started, the whole course should be completed within 7‐8 weeks as far as possible.

The appropriate dose of RT was also surveyed. A prospective randomized study demonstrated that the adequate dose for postoperative RT was 57.6 Gy for patients with low risk and 63 Gy for high risk head and neck cancer, with a fraction size of 1.8 Gy/day. Increasing the dose didn't significantly improve tumor control.^27^ Both RTOG 9501 and EORTC 22931 used doses of 60‐66 Gy with fraction size 2.0 Gy/day in postoperative RT settings for high‐risk squamous‐cell carcinoma of the head and neck.^2^, ^3^ Similarly in our analysis, doses less than 50 Gy or higher than 70 Gy, had a significantly detrimental impact on the outcome.

This study has several strengths over previous studies. First, our analysis included a large number of homogeneous patients with resected OSCC who then received adjuvant RT. It was more specific than the heterogeneous groups in previous historical data, which included all entities of head and neck cancer. Second, with the largest population of resected OSCC from the TCR database, the potential for confounding results due to the small size of the study group could be avoided. Third, by virtue of access to fuller information from the TCR including date and type of first recurrence, cancer status, and cause of death, the endpoints of LRFS, CSS, and DMFS could also be analyzed beyond OS.

There are some limitations for our study. First, the TCR data was from Taiwan, which thus is subject to geographic bias. Second, the TCR only recorded the first relapse data regardless of further failure after the first relapse, which would underestimate the actual relapse rate of all the patients in this data‐base analysis. Third, as in any previous database study, our study inevitably included existing confoundings, coding errors, selection bias, and missing data. However, these can be ameliorated with continued dedication to accurate and complete information input, to lower factors which may bias analysis, making the TCR database a valuable resource for such studies.^6^ Furthermore, some established variables, including smoking, alcohol consumption, betel nut chewing history, depth of invasion, lymphovascular infiltration, perineural infiltration as well as precise surgical or radiation technique, could only be accessible in TCR database after 2011. We chose not to incorporate these variables in current study because of small size with not enough follow‐up time, which could lead to insufficient statistical power. Future works will be done and will elucidate the further outcomes once the follow‐up time is long enough for incorporating all additional variables accessed since 2011.

## 5 CONCLUSIONS

Through the use of this large national cancer registry, patients’ gender, age, cancer subsites, tumor stage, histologic grade, margin, and ENE status were found to be associated prognostic factors in patients with resected OSCC receiving adjuvant radiotherapy. The waiting time from surgery to starting adjuvant RT did not show a significant impacts on survival, while RTT > 8 weeks and extreme RT dose (>70 Gy or ≤50 Gy) were independently identified as significant predictors for poor prognosis. These results suggest that effort should be made to ensure that patients with resected OSCC should complete their adjuvant RT course within 8 weeks, with sufficiently adequate doses to avoid potentially detrimental outcomes.

## CONFLICT OF INTEREST

None declared.
