# Supplementary material for: Adjuvant radiotherapy after curative surgery for oral cavity squamous cell carcinoma and treatment effect of timing and duration on outcome—A Taiwan Cancer Registry national database analysis
Source: Cancer Med. 2018 Jun 14;7(7):3073–83. doi: 10.1002/cam4.1611 (PMC6051157; doi:10.1002/cam4.1611)
Supplement: Supplementary file 1 [file CAM4-7-3073-s001.docx]

| **Table S1.** Univariate analysis of factors associated with each endpoints | | | | | | | | | | | | |
| --- | --- | --- | --- | --- | --- | --- | --- | --- | --- | --- | --- | --- |
|  |  | OS | |  | CSS | |  | LRFS | |  | DMFS | |
| **Patient Characteristics** |  | HR (95% CI) | P-value |  | HR (95% CI) | P-value |  | HR (95% CI) | P-value |  | HR (95% CI) | P-value |
| Sex |  |  | 0.109 |  |  | 0.464 |  |  | **0.012** |  |  | 0.284 |
| Male |  | Ref |  |  | Ref |  |  | Ref |  |  | Ref |  |
| Female |  | 0.90 (0.78-1.02) | 0.109 |  | 0.95 (0.82-1.09) | 0.465 |  | **0.78 (0.64-0.94)** | **0.013** |  | 0.86 (0.64-1.12) | 0.285 |
| Age |  |  | **<.001** |  |  | **0.003** |  |  | 0.066 |  |  | **0.011** |
| <40 |  | Ref |  |  | Ref |  |  | Ref |  |  | Ref |  |
| 40-49 |  | 0.96 (0.85-1.09) | 0.561 |  | 0.94 (0.83-1.08) | 0.391 |  | 1.00 (0.85-1.18) | 0.970 |  | 0.94 (0.75-1.19) | 0.595 |
| 50-59 |  | 0.94 (0.83-1.06) | 0.281 |  | **0.88 (0.77-1.00)** | **0.047** |  | 0.91 (0.78-1.07) | 0.238 |  | 0.82 (0.65-1.04) | 0.088 |
| 60-69 |  | 1.06 (0.93-1.21) | 0.377 |  | 0.94 (0.81-1.08) | 0.389 |  | **0.83 (0.69-1.00)** | **0.043** |  | **0.66 (0.50-0.87)** | **0.003** |
| >70 |  | **1.40 (1.20-1.63)** | **<.001** |  | 1.17 (0.98-1.38) | 0.081 |  | 0.85 (0.67-1.08) | 0.187 |  | 0.77 (0.55-1.08) | 0.143 |
| **Disease characteristics** |  |  |  |  |  |  |  |  |  |  |  |  |
| Site of disease |  |  | **0.010** |  |  | **0.038** |  |  | **<.001** |  |  | 0.882 |
| Buccal mucosa |  | Ref |  |  | Ref |  |  | Ref |  |  | Ref |  |
| Lip |  | 0.87 (0.68-1.09) | 0.246 |  | 0.87 (0.67-1.12) | 0.295 |  | 0.90 (0.65-1.20) | 0.489 |  | 0.70 (0.40-1.13) | 0.180 |
| Oral tongue |  | 1.05 (0.97-1.14) | 0.229 |  | 1.02 (0.93-1.12) | 0.648 |  | 0.96 (0.85-1.07) | 0.421 |  | 0.98 (0.83-1.16) | 0.823 |
| Gum |  | 0.98 (0.88-1.09) | 0.724 |  | 0.97 (0.86-1.09) | 0.614 |  | 0.91 (0.78-1.05) | 0.187 |  | 0.98 (0.79-1.21) | 0.832 |
| Floor of Mouth |  | 1.01 (0.84-1.19) | 0.925 |  | 0.87 (0.71-1.06) | 0.173 |  | **0.47 (0.33-0.64)** | **<.001** |  | 1.09 (0.77-1.51) | 0.605 |
| Hard palate |  | **1.34 (1.08-1.63)** | **0.006** |  | **1.30 (1.03-1.61)** | **0.024** |  | **1.33 (0.99-1.74)** | **0.047** |  | 0.95 (0.56-1.50) | 0.836 |
| Other parts of Mouth |  | **1.17 (1.03-1.32)** | **0.013** |  | 1.13 (0.99-1.30) | 0.072 |  | 1.16 (0.98-1.37) | 0.089 |  | 1.02 (0.78-1.32) | 0.886 |
| Pathologic AJCC stage |  |  | **<.001** |  |  | **<.001** |  |  | **<.001** |  |  | **<.001** |
| Stage1 |  | Ref |  |  | Ref |  |  | Ref |  |  | Ref |  |
| Stage2 |  | **1.33 (1.18-1.50)** | **<.001** |  | 1.03 (0.80-1.32) | 0.828 |  | 1.03 (0.80-1.33) | 0.827 |  | 1.91 (0.83-5.16) | 0.156 |
| Stage3 |  | **1.57 (1.36-1.81)** | **<.001** |  | **1.44 (1.16-1.82)** | **0.002** |  | 1.22 (0.97-1.55) | 0.104 |  | **4.91 (2.35-12.59)** | **<.001** |
| Stage4 |  | **1.62 (1.44-1.82)** | **<.001** |  | **2.45 (2.00-3.04)** | **<.001** |  | **1.44 (1.16-1.80)** | **0.001** |  | **11.78 (5.79-29.71)** | **<.001** |
| Grade |  |  | **<.001** |  |  | **<.001** |  |  | **<.001** |  |  | **<.001** |
| Well differentiated |  | Ref |  |  | Ref |  |  | Ref |  |  | Ref |  |
| Moderately differentiated |  | **1.29 (1.19-1.41)** | **<.001** |  | **1.29 (1.18-1.42)** | **<.001** |  | **1.14 (1.02-1.28)** | **0.022** |  | **1.88 (1.54-2.30)** | **<.001** |
| Poorly or Undifferentiated |  | **1.92 (1.72-2.15)** | **<.001** |  | **1.97 (1.74-2.23)** | **<.001** |  | **1.54 (1.32-1.81)** | **<.001** |  | **2.88 (2.25-3.68)** | **<.001** |
| Unknown |  | **1.40 (1.04-1.84)** | **0.019** |  | **1.53 (1.11-2.03)** | **0.006** |  | **1.47 (1.01-2.08)** | **0.034** |  | 1.12 (0.47-2.23) | 0.764 |
| **Table S1.** (continued) Univariate analysis of factors associated with each endpoints | | | | | | | | | | | | |
| Margin |  |  | **<.001** |  |  | **<.001** |  |  | **<.001** |  |  | **0.037** |
| Negative |  | Ref |  |  | Ref |  |  | Ref |  |  | Ref |  |
| Positive |  | **1.75 (1.58-1.92)** | **<.001** |  | **1.77 (1.58-1.97)** | **<.001** |  | **1.53 (1.32-1.78)** | **<.001** |  | **1.29 (1.01-1.63)** | **<.05** |
| ENE (2011-2015) |  |  | **<.001** |  |  | **<.001** |  |  | **<.001** |  |  | **<.001** |
| Negative |  | Ref |  |  | Ref |  |  | Ref |  |  | Ref |  |
| Positive |  | **2.60 (2.36-2.85)** | **<.001** |  | **2.91 (2.63-3.22)** | **<.001** |  | **2.11 (1.84-2.41)** | **<.001** |  | **3.65 (3.05-4.38)** | **<.001** |
| **Treatment parameters** |  |  |  |  |  |  |  |  |  |  |  |  |
| Treatment type |  |  | **<.001** |  |  | **<.001** |  |  | 0.532 |  |  | **<.001** |
| OP->RT |  | Ref |  |  | Ref |  |  | Ref |  |  | Ref |  |
| OP->CCRT |  | 1.41 (1.31-1.53) | **<.001** |  | **1.55 (1.42-1.69)** | **<.001** |  | 1.06 (0.96-1.17) | 0.293 |  | **2.72 (2.26-3.30)** | **<.001** |
| OP->RT+CT |  | 1.42 (1.20-1.66) | **<.001** |  | **1.52 (1.27-1.81)** | **<.001** |  | 1.09 (0.86-1.36) | 0.447 |  | **1.75 (1.15-2.56)** | **0.006** |
| CT->OP->RT |  | 1.50 (1.16-1.89) | **0.001** |  | **1.78 (1.36-2.28)** | **<.001** |  | 1.21 (0.85-1.66) | 0.259 |  | **2.22 (1.25-3.65)** | **0.004** |
| OP to RT interval (ORI) |  |  | **<.001** |  |  | **<.001** |  |  | **0.003** |  |  | 0.678 |
| 0-4 weeks |  | Ref |  |  | Ref |  |  | Ref |  |  | Ref |  |
| 4-5 weeks |  | **0.88 (0.80-0.98)** | **0.017** |  | 0.90 (0.80-1.00) | 0.056 |  | 0.95 (0.82-1.09) | 0.463 |  | 1.11 (0.90-1.37) | 0.324 |
| 5-6 weeks |  | **0.89 (0.80-0.98)** | **0.013** |  | **0.89 (0.80-0.99)** | **0.038** |  | 0.89 (0.78-1.02) | 0.095 |  | 1.07 (0.88-1.32) | 0.499 |
| 6-7 weeks |  | 0.95 (0.84-1.08) | 0.414 |  | 0.93 (0.81-1.07) | 0.307 |  | 1.03 (0.87-1.23) | 0.712 |  | 0.99 (0.75-1.29) | 0.917 |
| >7 weeks |  | 1.11 (0.99-1.24) | 0.064 |  | **1.14 (1.01-1.28)** | **0.037** |  | **1.18 (1.01-1.37)** | **0.037** |  | 0.96 (0.75-1.23) | 0.754 |
| RT treatment time (RTT) |  |  | **<.001** |  |  | **<.001** |  |  | **<.001** |  |  | **<.001** |
| 0-6 weeks |  | Ref |  |  | Ref |  |  | Ref |  |  | Ref |  |
| 6-7 weeks |  | **1.12 (1.02-1.23)** | **0.024** |  | **1.17 (1.05-1.31)** | **0.004** |  | 1.09 (0.95-1.25) | 0.205 |  | 1.44 (1.16-1.79) | **0.001** |
| 7-8 weeks |  | 1.41 (1.26-1.57) | **<.001** |  | **1.14 (0.98-1.32)** | **<.001** |  | **1.45 (1.24-1.68)** | **<.001** |  | 1.89 (1.49-2.41) | **<.001** |
| >8 weeks |  | **1.79 (1.59-2.02)** | **<.001** |  | **1.46 (1.25-1.70)** | **<.001** |  | **1.78 (1.51-2.11)** | **<.001** |  | 1.59 (1.20-2.12) | **0.001** |
| RT dose |  |  | **<.001** |  |  | **<.001** |  |  | **<.001** |  |  | **<.001** |
| 45-50 Gy |  | **2.38 (1.85-3.00)** | **<.001** |  | **2.34 (1.76-3.05)** | **<.001** |  | **1.52 (0.98-2.24)** | **0.047** |  | 1.56 (0.70-2.99) | 0.219 |
| 50-60 Gy |  | Ref |  |  | Ref |  |  | Ref |  |  | Ref |  |
| 60-70 Gy |  | **1.39 (1.28-1.51)** | **<.001** |  | **1.46 (1.33-1.61)** | **<.001** |  | **1.24 (1.11-1.39)** | **<.001** |  | **1.87 (1.55-2.26)** | **<.001** |
| >70 Gy |  | **2.05 (1.76-2.37)** | **<.001** |  | **2.25 (1.91-2.63)** | **<.001** |  | **1.95 (1.58-2.38)** | **<.001** |  | **2.66 (1.92-3.64)** | **<.001** |

Abbreviations: AJCC, American joint committee on Cancer; ENE, extra-nodal extension; RT, radiotherapy; CCRT, concurrent chemoradiation therapy; CT, chemotherapy; OP, operation; ORI, OP-RT interval; RTT, RT treatment time; HR, hazard ratio; CI, confidence interval; OS, overall survival; LRFS, local-regional free survival; CSS, cancer-specific survival; DMFS, distant-metastasis free survival

**Table S2.** Relapse pattern of the patients (n=8,986; 2007-2015)

| Relapse pattern | No. of cases | Percent |
| --- | --- | --- |
| No | 6069 | 67.5% |
| Local | 695 | 7.7% |
| Regional | 787 | 8.8% |
| Distant metastasis | 593 | 6.6% |
| Local + regional | 120 | 1.3% |
| Local/regional + metastasis | 215 | 2.4% |
| Residual tumor | 507 | 5.6% |

| **Table S3.** Multivariate analysis of factors including ENE associated with each endpoints (since 2011) | | | | | | | | | | | | |
| --- | --- | --- | --- | --- | --- | --- | --- | --- | --- | --- | --- | --- |
|  |  | OS | |  | CSS | |  | LRFS | |  | DMFS | |
| **Patient Characteristics** |  | HR (95% CI) | P-value |  | HR (95% CI) | P-value |  | HR (95% CI) | P-value |  | HR (95% CI) | P-value |
| Sex |  |  |  |  |  |  |  |  |  |  |  |  |
| Male |  | Ref |  |  | Ref |  |  | Ref |  |  | Ref |  |
| Female |  | 0.90 (0.75-1.08) | 0.270 |  | 1.00 (0.82-1.20) | 0.962 |  | 0.77 (0.57-1.00) | 0.063 |  | 0.96 (0.67-1.35) | 0.838 |
| Age |  |  |  |  |  |  |  |  |  |  |  |  |
| <40 |  | Ref |  |  | Ref |  |  | Ref |  |  | Ref |  |
| 40-49 |  | 0.97 (0.81-1.16) | 0.725 |  | 0.95 (0.78-1.15) | 0.576 |  | 1.14 (0.89-1.48) | 0.296 |  | 1.05 (0.77-1.48) | 0.751 |
| 50-59 |  | 0.98 (0.82-1.17) | 0.804 |  | 0.91 (0.76-1.11) | 0.350 |  | 1.10 (0.87-1.43) | 0.432 |  | 0.87 (0.63-1.21) | 0.392 |
| 60-69 |  | 1.11 (0.92-1.34) | 0.288 |  | 1.03 (0.84-1.27) | 0.781 |  | 0.94 (0.72-1.25) | 0.680 |  | 0.83 (0.58-1.20) | 0.306 |
| >70 |  | **1.55 (1.24-1.94)** | **<.001** |  | 1.24 (0.96-1.60) | 0.096 |  | 1.07 (0.76-1.50) | 0.712 |  | 1.01 (0.63-1.61) | 0.954 |
| **Disease characteristics** |  |  |  |  |  |  |  |  |  |  |  |  |
| Site of disease |  |  |  |  |  |  |  |  |  |  |  |  |
| Buccal mucosa |  | Ref |  |  | Ref |  |  | Ref |  |  | Ref |  |
| Lip |  | 1.01 (0.72-1.36) | 0.964 |  | 1.07 (0.74-1.48) | 0.717 |  | 0.78 (0.47-1.23) | 0.318 |  | 1.13 (0.59-1.96) | 0.680 |
| Oral tongue |  | **1.13 (1.00-1.27)** | **0.045** |  | 1.11 (0.97-1.26) | 0.123 |  | 1.01 (0.86-1.18) | 0.926 |  | 1.11 (0.88-1.40) | 0.371 |
| Gum |  | 1.04 (0.90-1.21) | 0.567 |  | 1.05 (0.89-1.23) | 0.548 |  | 0.99 (0.80-1.21) | 0.912 |  | 1.14 (0.85-1.51) | 0.374 |
| Floor of Mouth |  | 1.01 (0.79-1.27) | 0.959 |  | 0.85 (0.64-1.12) | 0.263 |  | **0.53 (0.34-0.79)** | **0.004** |  | 0.99 (0.61-1.54) | 0.963 |
| Hard palate |  | **1.45 (1.09-1.91)** | **0.009** |  | **1.46 (1.05-1.97)** | **0.018** |  | **1.84 (1.26-2.59)** | **0.001** |  | 1.10 (0.52-2.05) | 0.787 |
| Other parts of Mouth |  | 1.11 (0.93-1.32) | 0.252 |  | 1.03 (0.84-1.24) | 0.804 |  | 0.99 (0.77-1.27) | 0.958 |  | 1.05 (0.73-1.48) | 0.777 |
| Pathologic AJCC stage |  |  |  |  |  |  |  |  |  |  |  |  |
| Stage1 |  | Ref |  |  | Ref |  |  | Ref |  |  | Ref |  |
| Stage2 |  | 1.05 (0.77-1.44) | 0.780 |  | 1.03 (0.72-1.51) | 0.860 |  | 1.01 (0.69-1.48) | 0.978 |  | 2.84 (0.77-18.26) | 0.172 |
| Stage3 |  | **1.37 (1.04-1.84)** | **0.030** |  | **1.36 (0.98-1.93)** | **0.072** |  | 1.23 (0.88-1.77) | 0.235 |  | **6.19 (1.92-37.88)** | **0.012** |
| Stage4 |  | **1.91 (1.47-2.54)** | **<.001** |  | **2.09 (1.54-2.93)** | **<.001** |  | 1.37 (0.99-1.94) | 0.064 |  | **12.38 (3.93-75.07)** | **<.001** |
| Grade |  |  |  |  |  |  |  |  |  |  |  |  |
| Well differentiated |  | Ref |  |  | Ref |  |  | Ref |  |  | Ref |  |
| Moderately differentiated |  | **1.26 (1.11-1.43)** | **<.001** |  | **1.25 (1.09-1.45)** | **0.002** |  | **1.20 (1.01-1.42)** | **0.040** |  | **1.61 (1.22-2.14)** | **<.001** |
| Poorly or Undifferentiated |  | **1.73 (1.47-2.04)** | **<.001** |  | **1.76 (1.47-2.10)** | **<.001** |  | **1.56 (1.23-1.96)** | **<.001** |  | **2.51 (1.80-3.53)** | **<.001** |
| Unknown |  | 0.85 (0.38-1.63) | 0.657 |  | 0.88 (0.37-1.77) | 0.750 |  | 1.72 (0.75-3.43) | 0.157 |  | 1.42 (0.23-4.81) | 0.634 |
| **Table S3.** (continued) Multivariate analysis of factors including ENE associated with each endpoints (since 2011) | | | | | | | | | | | | |
| Margin |  |  |  |  |  |  |  |  |  |  |  |  |
| Negative |  | Ref |  |  | Ref |  |  | Ref |  |  | Ref |  |
| Positive |  | **1.76 (1.53-2.01)** | **<.001** |  | **1.76 (1.52-2.04)** | **<.001** |  | **1.55 (1.25-1.91)** | **<.001** |  | 1.23 (0.89-1.66) | 0.195 |
| ENE (2011-2015) |  |  |  |  |  |  |  |  |  |  |  |  |
| Negative |  | Ref |  |  | Ref |  |  | Ref |  |  | Ref |  |
| Positive |  | **2.21 (1.98-2.46)** | **<.001** |  | **2.35 (2.09-2.64)** | **<.001** |  | **1.94 (1.66-2.27)** | **<.001** |  | **2.21 (1.81-2.71)** | **<.001** |
| **Treatment parameters** |  |  |  |  |  |  |  |  |  |  |  |  |
| Treatment type |  |  |  |  |  |  |  |  |  |  |  |  |
| OP->RT |  | Ref |  |  | Ref |  |  | Ref |  |  | Ref |  |
| OP->CCRT |  | 0.90 (0.79-1.02) | 0.094 |  | 0.95 (0.83-1.11) | 0.537 |  | **0.80 (0.68-0.96)** | **0.015** |  | **1.40 (1.05-1.89)** | **0.025** |
| OP->RT+CT |  | 0.89 (0.68-1.13) | 0.342 |  | 0.89 (0.67-1.17) | 0.418 |  | 0.90 (0.63-1.26) | 0.547 |  | 0.91 (0.48-1.62) | 0.751 |
| CT->OP->RT |  | 0.96 (0.61-1.43) | 0.833 |  | 1.09 (0.67-1.67) | 0.727 |  | 1.03 (0.58-1.71) | 0.906 |  | 0.60 (0.14-1.68) | 0.399 |
| OP to RT interval (ORI) |  |  |  |  |  |  |  |  |  |  |  |  |
| 0-4 weeks |  | Ref |  |  | Ref |  |  | Ref |  |  | Ref |  |
| 4-5 weeks |  | 0.94 (0.82-1.09) | 0.431 |  | 0.93 (0.79-1.09) | 0.346 |  | 1.01 (0.83-1.23) | 0.924 |  | 1.15 (0.88-1.52) | 0.319 |
| 5-6 weeks |  | 0.95 (0.83-1.08) | 0.433 |  | 0.96 (0.83-1.11) | 0.573 |  | 1.00 (0.83-1.21) | 0.982 |  | 1.10 (0.86-1.43) | 0.457 |
| 6-7 weeks |  | 1.09 (0.91-1.31) | 0.352 |  | 1.03 (0.84-1.26) | 0.763 |  | 1.00 (0.76-1.30) | 0.997 |  | 0.83 (0.55-1.24) | 0.378 |
| >7 weeks |  | 1.17 (0.99-1.39) | 0.062 |  | **1.20 (1.00-1.45)** | **0.050** |  | 1.08 (0.84-1.38) | 0.561 |  | 0.97 (0.66-1.41) | 0.873 |
| RT treatment time (RTT) |  |  |  |  |  |  |  |  |  |  |  |  |
| 0-6 weeks |  | Ref |  |  | Ref |  |  | Ref |  |  | Ref |  |
| 6-7 weeks |  | 0.91 (0.77-1.08) | 0.296 |  | 0.90 (0.75-1.10) | 0.302 |  | 0.96 (0.76-1.23) | 0.754 |  | 0.90 (0.63-1.31) | 0.577 |
| 7-8 weeks |  | 1.04 (0.86-1.26) | 0.691 |  | 1.07 (0.87-1.32) | 0.535 |  | **1.30 (1.00-1.71)** | **0.054** |  | 1.15 (0.77-1.72) | 0.495 |
| >8 weeks |  | **1.38 (1.12-1.70)** | **0.003** |  | **1.36 (1.07-1.72)** | **0.011** |  | **1.55 (1.15-2.10)** | **0.004** |  | 1.01 (0.63-1.61) | 0.955 |
| RT dose |  |  |  |  |  |  |  |  |  |  |  |  |
| 45-50 Gy |  | **2.52 (1.76-3.50)** | **<.001** |  | **2.42 (1.58-3.54)** | **<.001** |  | **2.04 (1.10-3.47)** | **0.014** |  | 1.68 (0.51-4.10) | 0.315 |
| 50-60 Gy |  | Ref |  |  | Ref |  |  | Ref |  |  | Ref |  |
| 60-70 Gy |  | 1.14 (0.98-1.33) | 0.102 |  | 1.14 (0.96-1.35) | 0.149 |  | 1.17 (0.95-1.45) | 0.157 |  | 1.18 (0.86-1.65) | 0.323 |
| >70 Gy |  | **1.31 (1.02-1.66)** | **0.033** |  | **1.35 (1.03-1.76)** | **0.028** |  | **1.42 (1.00-2.00)** | **0.047** |  | 1.36 (0.80-2.25) | 0.244 |

Abbreviations: AJCC, American joint committee on Cancer; ENE, extra-nodal extension; RT, radiotherapy; CCRT, concurrent chemoradiation therapy; CT, chemotherapy; OP, operation; ORI, OP-RT interval; RTT, RT treatment time; HR, hazard ratio; CI, confidence interval; OS, overall survival; LRFS, local-regional free survival; CSS, cancer-specific survival; DMFS, distant-metastasis free survival
